# Characterization of Bacterial Microbiota Composition in Healthy and Diarrheal Early-Weaned Tibetan Piglets

**DOI:** 10.3389/fvets.2022.799862

**Published:** 2022-02-23

**Authors:** Qinghui Kong, Wenqian Zhang, Miao An, Muhammad Fakhar-e-Alam Kulyar, Zhenda Shang, Zhankun Tan, Yefen Xu, Jiakui Li, Suozhu Liu

**Affiliations:** ^1^College of Animal Science, Tibet Agricultural and Animal Husbandry University, Linzhi, China; ^2^College of Veterinary Medicine, Huazhong Agricultural University, Wuhan, China; ^3^Tibetan Plateau Feed Processing Engineering Research Center, Linzhi, China

**Keywords:** microbial diversity, bacteria, 16S rRNA, Tibetan piglets, diarrhea

## Abstract

The occurrence of diarrhea in Tibetan piglets is highly notable, but the microorganisms responsible are yet unclear. Its high incidence results in serious economic losses for the Tibetan pig industry. Moreover, the dynamic balance of intestinal microflora plays a crucial role in maintaining host health, as it is a prime cause of diarrhea. Therefore, the present study was performed to analyze the characteristics of bacterial microbiota structure in healthy, diarrheal and treated weaned piglets in Tibet autonomous region for providing a theoretical basis to prevent and control diarrhea. The study was based on the V3–V4 region of the 16S rRNA gene and gut microbiota functions following the metagenome analysis of fresh fecal samples (*n* = 5) from different groups. The Shannon and Simpson indices differed substantially between diarrheal and treated groups (*p* < 0.05). According to our findings, the beta diversities, especially between healthy and diarrheal groups, were found different. Firmicutes, Bacteroidetes and Proteobacteria were the dominant phyla in three groups. Furthermore, the abundance of Fusobacteria in the diarrheal group was higher than the other groups. The dominant genera in the diarrheal group were *Fusobacterium, Butyricimonas, Sutterella, Peptostreptococcus, and Pasteurella*. Moreover, *Lactobacillus, Megasphaera* and *Clavibacter* were distinctly less abundant in this group. It is noteworthy that the specific decrease in the abundance of pathogenic bacteria after antibiotic treatment in piglets was noticed, while the level of *Lactobacillus* was evidently increased. In conclusion, fecal microbial composition and structure variations were discovered across the three groups. Also, the ecological balance of the intestinal microflora was disrupted in diarrheal piglets. It might be caused by a reduction in the relative number of beneficial bacteria and an increase in the abundance of pathogenic bacteria. In the context of advocating for non-resistant feeding, we suspect that the addition of probiotics to feed may prevent early-weaning diarrhea in piglets. Moreover, our findings might help for preventing diarrhea in weaned Tibetan piglets with a better understanding of microbial population dynamics.

## Introduction

The Tibetan pig is a valuable indigenous specie, as it is the only one that can survive in China's high altitude. Tibetan pigs are mainly found in semi-grassland and semi-farmland regions of Tibet (1). Under long-term harsh environmental conditions, Tibetan pigs have developed resistance against cold and diseases by developing different characteristics. These characteristics have made them indispensable for pig production in the plateau (2).

Early weaning is often used in intensive pig production, both at home and on farms (3). Meanwhile, weaning is an important turning point for piglet's growth to reduce the rate of vertical disease transmission and helps in the overall improvement of a pig farm. Conversely, earlier weaning caused psychological, environmental and nutritional stress in piglets, which induced diarrhea, dystrophia and slow growth (4) resulting in significant economic losses for the pig industry. Studies have argued that the diarrhea of weaned piglets is caused by infection with multiple pathogenic factors (bacteria, virus, etc.) and the intestinal dysfunction of piglets (5). Also, the imperfect immune system of the piglets, environmental changes, dietary changes, and improper feeding methods are conducive to the invasion of pathogenic strains, e.g., *Escherichia coli* (6).

The dynamic balance of intestinal microbiota plays an important role in the immune regulation of animals (7). Normal intestinal microbiota can stimulate the animal intestinal immune system by improving the intestinal self-recognition and immune ability of different bacteria. The intestine also serves as a barrier that can reduce the probability of host infection. Furthermore, weaning stress has disrupted the natural gut balance, reducing helpful microorganisms (8).

Due to the harsh cultural environment and intensive breeding strategies, Tibetan piglets frequently suffer from diarrhea after weaning. These factors are significantly decreasing the production performance and economics. The present study was performed to analyze the microbial diversity of different bacterial strains in healthy and diarrheal Tibetan piglets in Nyingchi, Tibet autonomous region, to investigate the etiology of diarrhea and develop a theoretical framework for it's prevention and treatment.

## Materials and Methods

### Animal Feeding and Sample Collection

The experimental animals for this study were taken from five healthy sows (The sows were raised at Tibetan Pig Collaborative Research Center of Tibet Agriculture and Animal Husbandry University) maintained at similar conditions. The sows gave birth on the same day. Tibetan piglets and sows were bred together in a farrowing house (the temperature of the farrowing house was ~21°C, and the farrowing bed was strictly cleaned and disinfected). The feed was given to the piglets when they were 3 weeks old. At the age of 6 weeks, healthy Tibetan piglets were weaned and transferred to the nursery house (the temperature of the nursery house was 16°C).

Hermann-Bank (9) test criteria were used to determine healthy and diarrheal Tibetan piglets. The feces of healthy Tibetan piglets (piglets without any clinical symptoms) were granular or stripe-shaped for more than 2 days. While the feces of diarrheal piglets were thin and unformed for more than 2 days. The fecal samples of healthy and diarrheal piglets (the diarrheal early-weaned Tibetan piglets not birthed by the same sow) were simultaneously collected. All samples were transferred from the ranch to the laboratory using a vehicle-mounted refrigerator (−15°C). Then the samples were stored at −20°C for further evaluation. The marked diarrheal Tibetan piglets were treated with 1 mL of Gentamycin (4%) sulfate through intra muscular route (HuaXu Company, China, Product number: 17925752842). Five fecal samples from healthy piglets (group A; marked as A1, A2, A3, A4, and A5), five fecal samples from diarrheal piglets (group B; marked as B1, B2, B3, B4, and B5), and five fecal samples from post-treatment piglets (group C; marked as C1, C2, C3, C4, and C5) were selected.

### DNA Extraction

The microbial DNA was extracted from 15 fecal samples of piglets using QIAamp Fast DNA Stool Mini Kit (QIAGEN, Hilden, Germany) as per the manufacturer's recommendations. The concentration and quality of DNA were detected with a nucleic acid detector (Nanodrop, Thermo Scientific NC2000, USA) and 1.2% agarose gel electrophoresis, respectively.

### 16S rRNA Amplification

The standard bacteria V3–V4 hypervariable region gene PCR primers (forward primer: ACT CCT ACG GGA GGC AGC A; reverse primer: GGA CTA CHV GGG TWT CTA AT) were used. AxyPrep DNA Gel Extraction Kit (Axygen, CA, USA) and the 2% agarose gel electrophoresis were used for target fragment recovery and evaluation of PCR amplification product. Quant-iT PicoGreen dsDNA Assay Kit (Invitrogen, Waltham, Massachusetts, USA) was used to detect the recovered PCR products. Moreover, TruSeq Nano DNA Low Throughput Library Prep Kit (Illumina, CA, USA) was implied for sequence library construction. Amplified products' sequence ends were repaired by End Repair Mix2. PCR amplification was carried out to enrich the sequencing library template, and the library enrichment product was purified again via BECKMAN AMPure XP Beads. The library's final fragment-selection and purification were performed using 2% agarose gel electrophoresis.

The quality of libraries was examined on Agilent Bioanalyzer using Agilent High Sensitivity DNA Kit before sequencing procedure. The libraries with only one peak signal and no linker signal were considered for the process. Moreover, the libraries were quantified using Quant-iT PicoGreen dsDNA Assay Kit on Promega QuantiFluor fluorescence quantification system. The qualified library concentration was more than 2 nM. These qualified libraries were gradient diluted and mixed in proportion according to required sequencing. The MiSeq Reagent Kit V3 (600 cycles) was used to perform 2 × 300 bp paired-end sequencing on the MiSeq sequencing machine after the mixed libraries were denatured into single strands by sodium hydroxide.

### Sequence Data Processing and Statistical Analysis

Sequences analysis was established as operational taxonomic units (OTUs) via Uclust with over 97% similarity (10). The highest abundant sequence in each OUT was selected as the representative sequence (11). Then, OTUs were taxonomically classified and grouped by comparing with those in the Unite database (12). Micro microflora's richness and evenness index was calculated using the measurement indexes (Chao1, ACE, Shannon, and Simpson). Beta diversity based on the weighted UniFrac distance matrices were calculated with QIIME (Version 1.7.0), while the Cluster analysis was preceded by principal coordinate analysis (PCA) (13). The metastatic statistical algorithm was used to analyze the discrepancy in microbial communities between groups at the phylum and genus levels (14). The heat map was created via R software (v3.0.3), and all the data were evaluated statistically by one-way analysis of variance through SPSS 20.0 software (SPSS Inc., Chicago, Illinois 60606-6307, USA).

## Results

### Sequencing Results and OTU Cluster's Statistical Analysis

**Figure 1:**
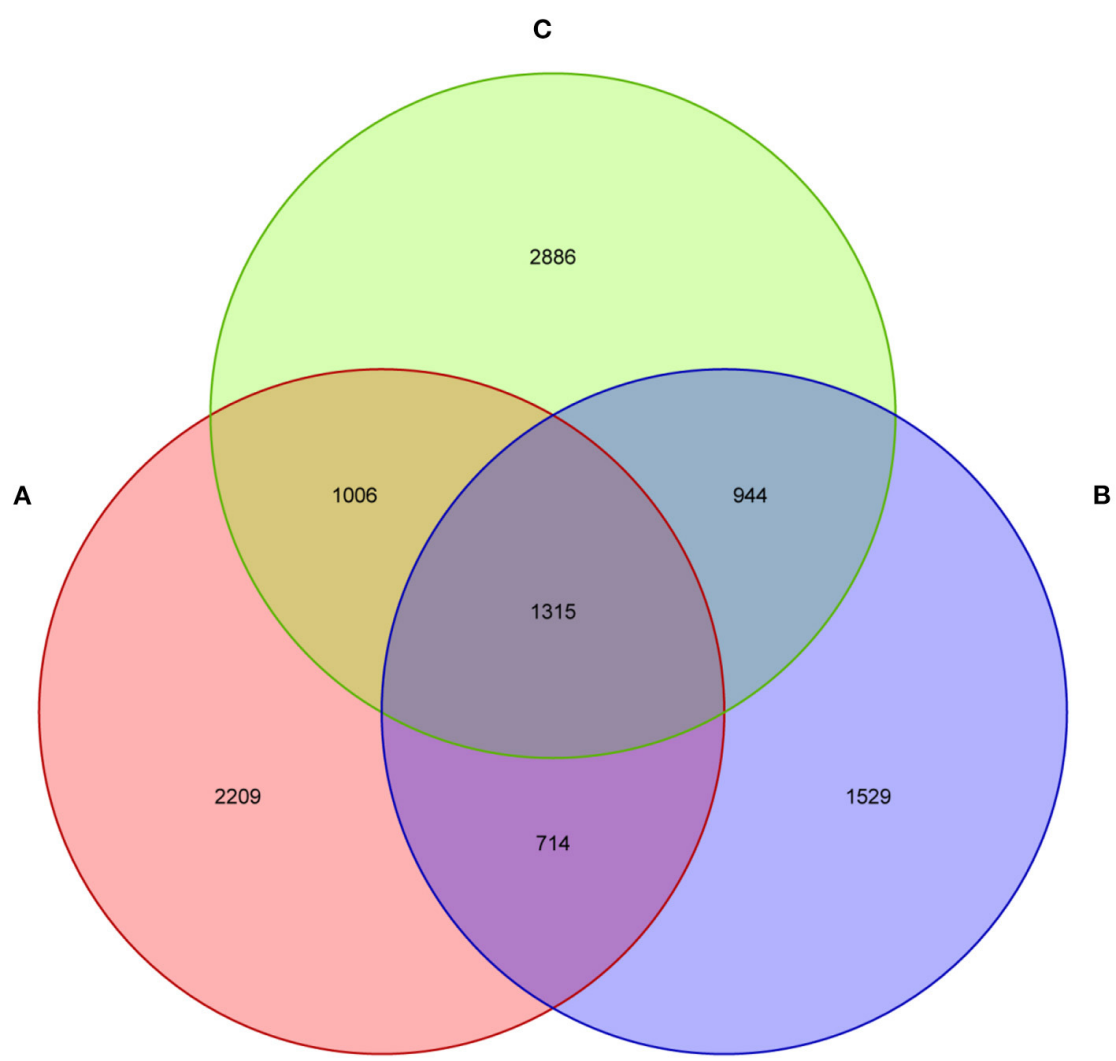
The fecal microflora of weaned piglets, analysed by a Venn diagram. **(A)** Healthy piglets; **(B)** Diarrheal piglets; **(C)** Treated piglets.

The current study subjected 15 fecal samples collected from Tibetan piglets to the high-throughput sequencing analysis. After optimizing the preliminary data, a total of 413,584, 427,913, and 408,109 high-quality valid sequences were obtained from the A, B and C groups, respectively. As shown in the dilution curve of species observation index, with the deepening test depth, its slope gradually decreased and reached the plateau stage. This finding indicated that the sequencing quantity of the samples was saturated, and the majority of bacteria were covered (Supplementary Figure 1). The sequences were established at the phylum, class, order, family, genus and species levels as OTUs via Uclust with over 97% similarity (Supplementary Figure 1). The three groups shared 1,315 bacteria species, as found by Venn map/diagram analysis (Figure 1). The diarrheal piglets showed 1,529 common bacteria species, which were not found in the healthy and antimicrobial-treated piglets. A total of 2,209 bacteria species were found to be common among the healthy piglets.

### Effects of Microbial Community Diversity

**Figure 2:**
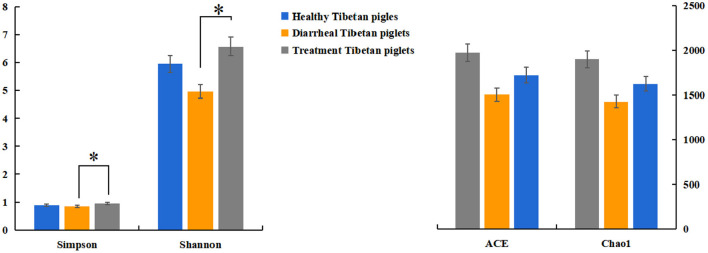
Diversity indices of the fecal microbiota in different Tibetan piglets. Chao1, ACE, Shannon, and Simpson indices were used to evaluate the alpha diversity of the fecal microbiota. The results were evaluated through one-way ANOVA. All of the data represent means ± SD. *p < 0.05.

**Figure 3:**
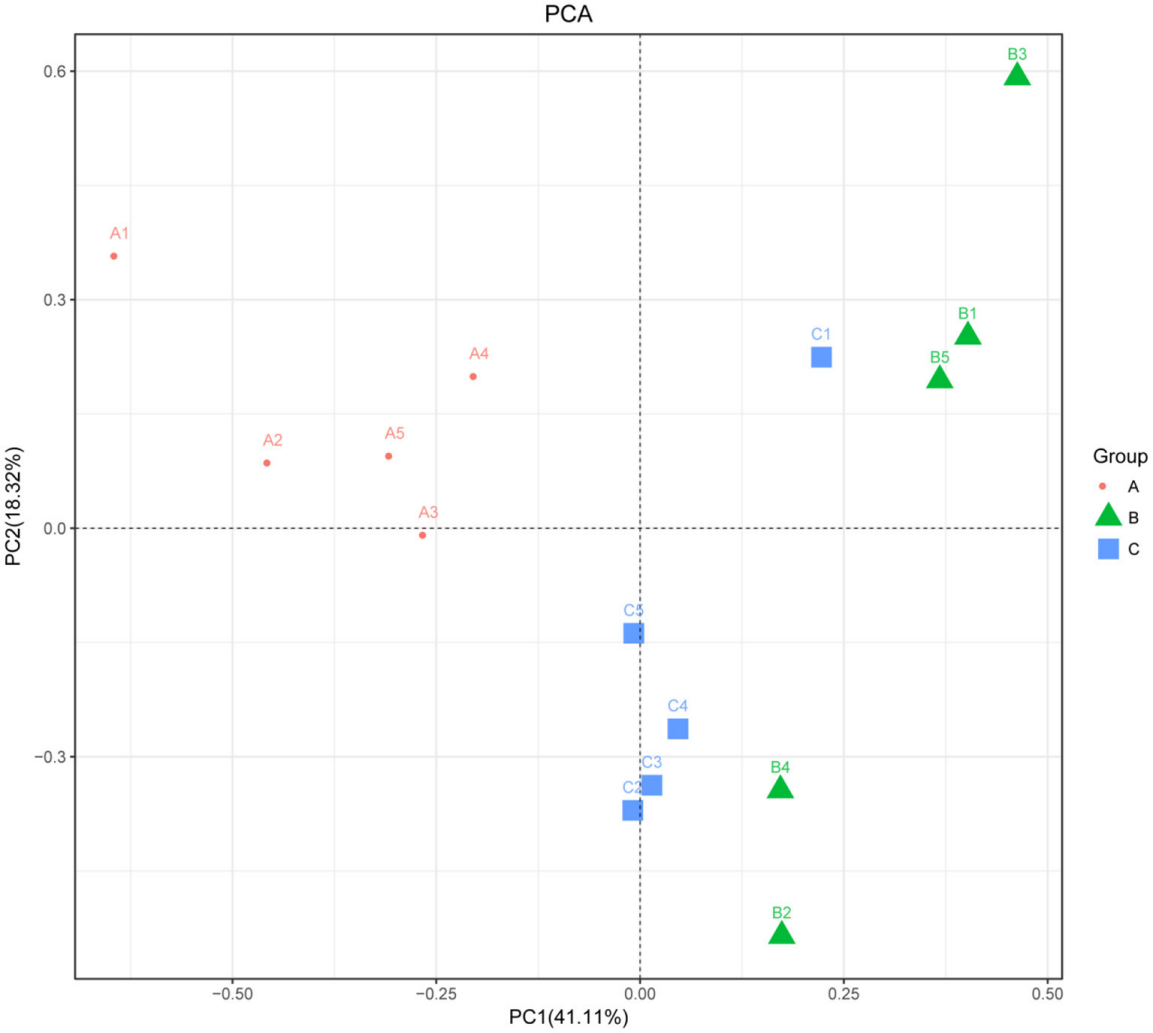
Principal component analysis of the fecal microbiota. PCA map based on Euclidean distance. Each point indicates one sample. The distance of the two points indicates the difference in fecal microbiota. A: Healthy piglets; B: Diarrheal piglets; C: Treated piglets.

The alpha diversity of fecal microbiota was evaluated by using Chao1, ACE, Shannon and Simpson. The Simpson and Shannon index demonstrated that there was no striking difference in the micro microflora abundance between group A (0.887, 5.962) and B (0.834, 4.97) (*p* > 0.05) (Figure 2), whereas C (0.945, 6.582) group was significantly higher than that of the B group (*p* < 0.05) (Figure 2). The ACE and Chao1 indices showed that A group and C group had higher richness than B group, whereas no striking difference in the microflora richness was noticed among the three groups (*p* > 0.05) (Figure 2). Specifically, The Chao1 index amounted to 1,625.74, 1,427.63, and 1,899.73 in groups A, B, and C, while the ACE index reached 1,721.43, 1,504.51, and 1,972.87 in groups A, B, and C, respectively. However, significant differences were found in the microbial community structure by principal component analysis (PCA) in different groups, especially among healthy piglets, as compared with other two groups (Figure 3).

### Composition Analysis of the Microbial Community Structure in Different Groups

**Figure 4:**
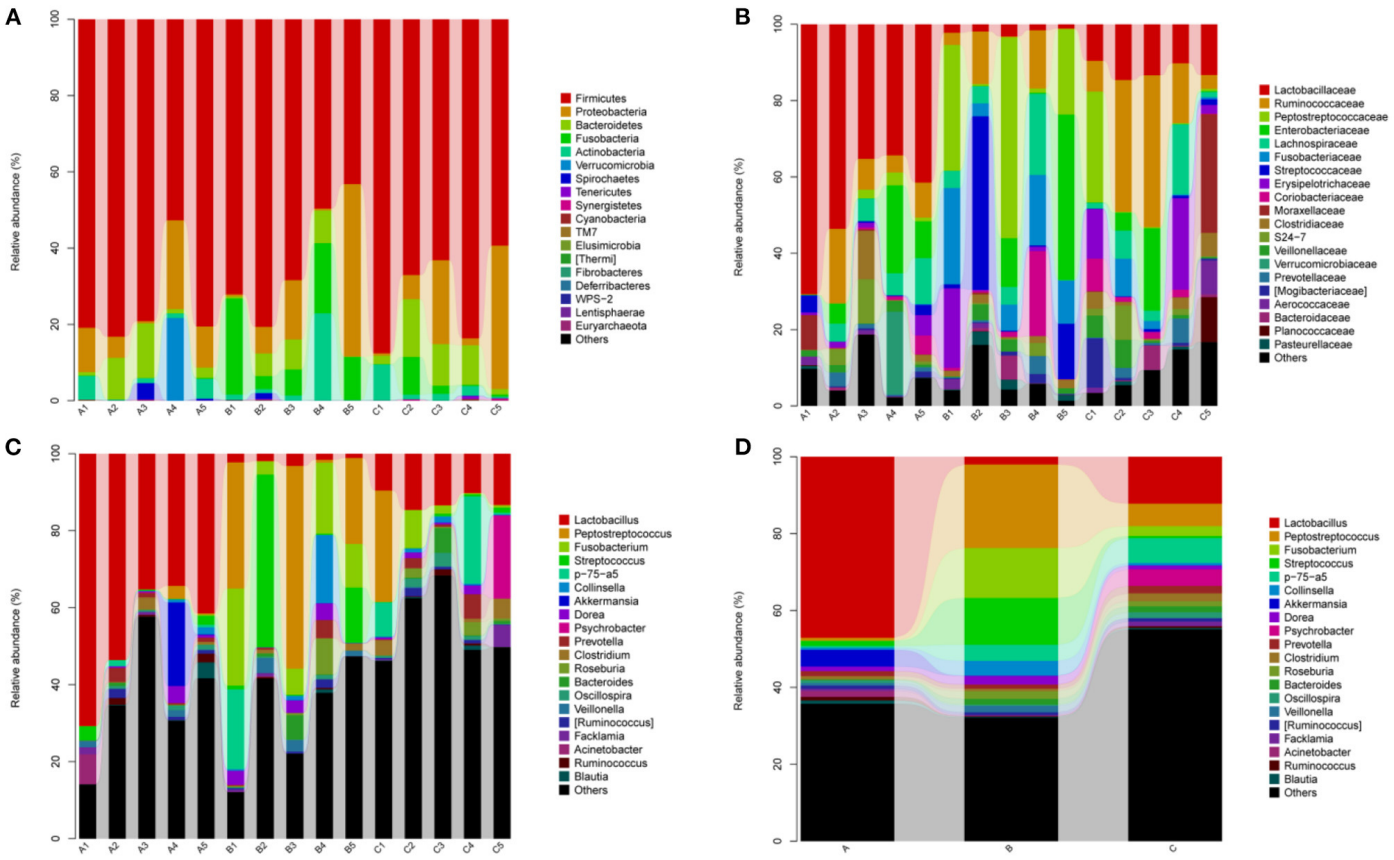
Relative abundance of gut bacterial taxa among different groups. **(A)** Phylum level; **(B)** Family level; **(C)** Genus level; **(D)** Taxonomic assignment at genus level. A1–A5: Healthy piglets; B1–B5: Diarrheal piglets; C1–C5: Treatment piglets.

The bacterial community in the three groups were assessed at different taxonomical levels. Firmicutes (75.28 ± 12.70% in group A, 62.78 ± 15.75% in group B, 72.16 ± 12.65% in group C) and Proteobacteria (10.36 ± 8.48% in group A, 13.76 ± 18.62% in group B, 13.66 ± 15.87% in group C) were dominant in all samples at the phylum level (Figure 4A). Other phyla, including Bacteroidetes and Actinobacteria, presented a lower abundance (<8% of all samples) (Figure 4A). Interestingly, Fusobacteria in group B (13.02 ± 8.82%) was higher as compared to group A (0.08 ± 0.13%) and group C (2.58 ± 4.16%). *Peptostreptococcaceae* (21.92 ± 22.13%), *Enterobacteriaceae* (11.32 ± 18.75%), *Streptococcaceae* (12.32 ± 19.45%), *Collinsella* (3.86 ± 7.75%), *Dorea* (2.26 ± 2.09%) were predominant in the B group, whereas *Psychrobacter* (4.30 ± 9.61%) and *Clostridium* (2.04 ± 2.34%) in the C group at the genus level (Figures 4C,D). In addtion, *Lactobacillus* (47.10 ± 15.31% in group A, 2.00 ± 0.78% in group B, 12.22 ± 2.18% in group C) and *Akkermansia* (4.38 ± 9.68% in A group) were predominant bacteria genera. The relative abundance of genera *Prevotella, Roseburia* and *Bacteroides* were <2% in all samples (Figures 4C,D).

**Figure 5:**
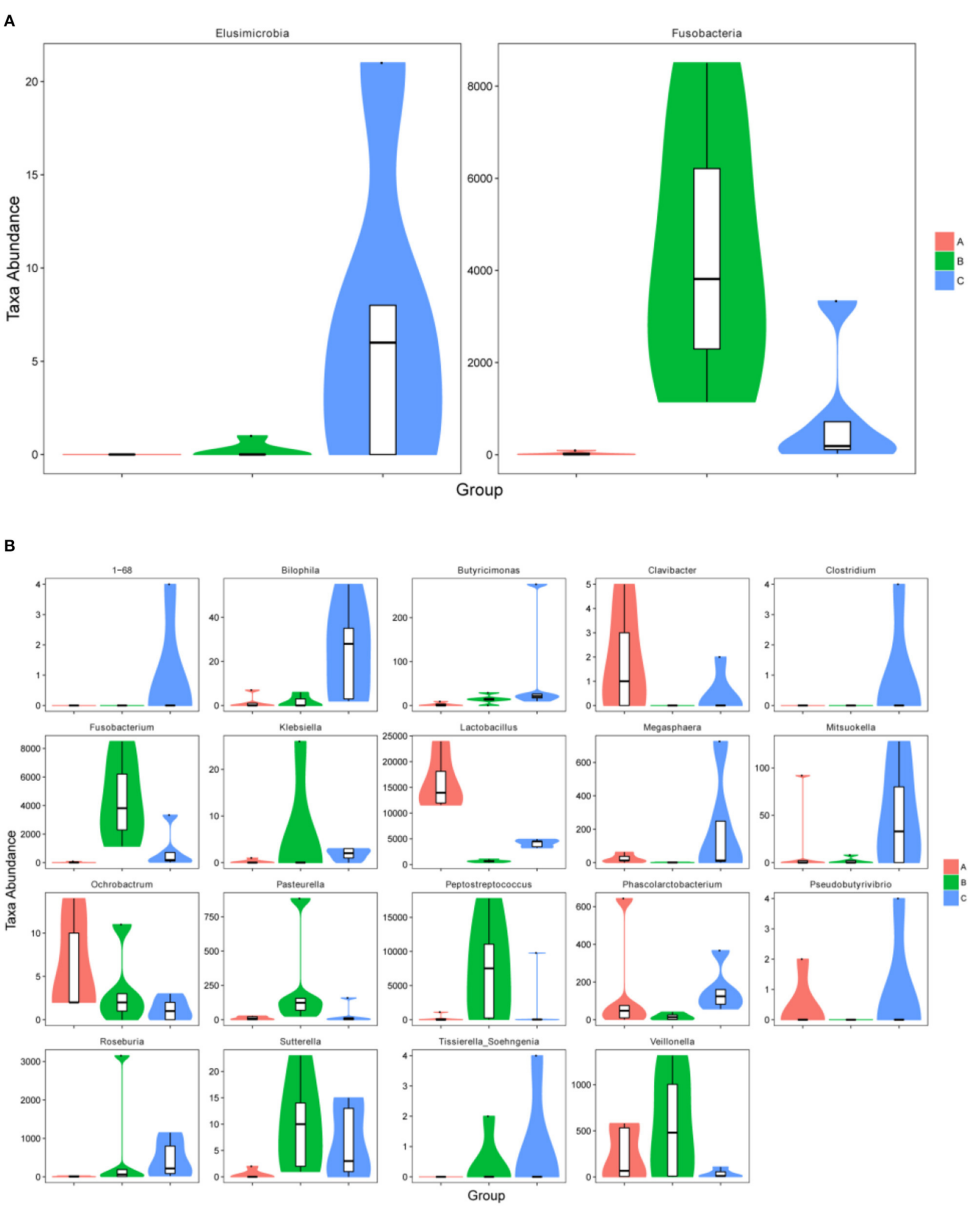
The metastatic composition of microbial diversity. **(A)** Microbial diversity at phylum level, **(B)** microbial diversity at genus level. A: Healthy piglets; B: Diarrheal piglets; C: Treated piglets.

The relative abundance of Fusobacteria in group B was significantly higher as compared to group A (*p* < 0.01) and group C (*p* < 0.05) at the phylum level (Figure 5A). The abundance of Elusimicrobia (*p* < 0.05) in the C group was significantly higher than group A and group B (Figure 5A). Furthermore, *Fusobacterium, Butyricimonas, Sutterella, Peptostreptococcus, Pasteurella* and *Veillonella* were the most abundant genus in group B, which were significantly higher than in other groups (*p* < 0.05) (Figure 5B). In contrast, *Megasphaera* and *Clavibacter* were less abundant in diarrheal piglets than healthy piglets (*p* < 0.05) (Figure 5B). *Lactobacillus* in the A group was significantly higher (*p* < 0.01) than in the B and C groups, whereas the abundance in group C was also significantly higher than that in group B (*p* < 0.01) (Figure 5B). Moreover, the relative abundance of *Klebsiella, Bilophila, Roseburia, 1–68, Clostridium, Sutterella* and *Tissierella_Soehngenia* in group C (*p* < 0.05) were significantly higher than in group A at the genus level (Figure 5B).

**Figure 6:**
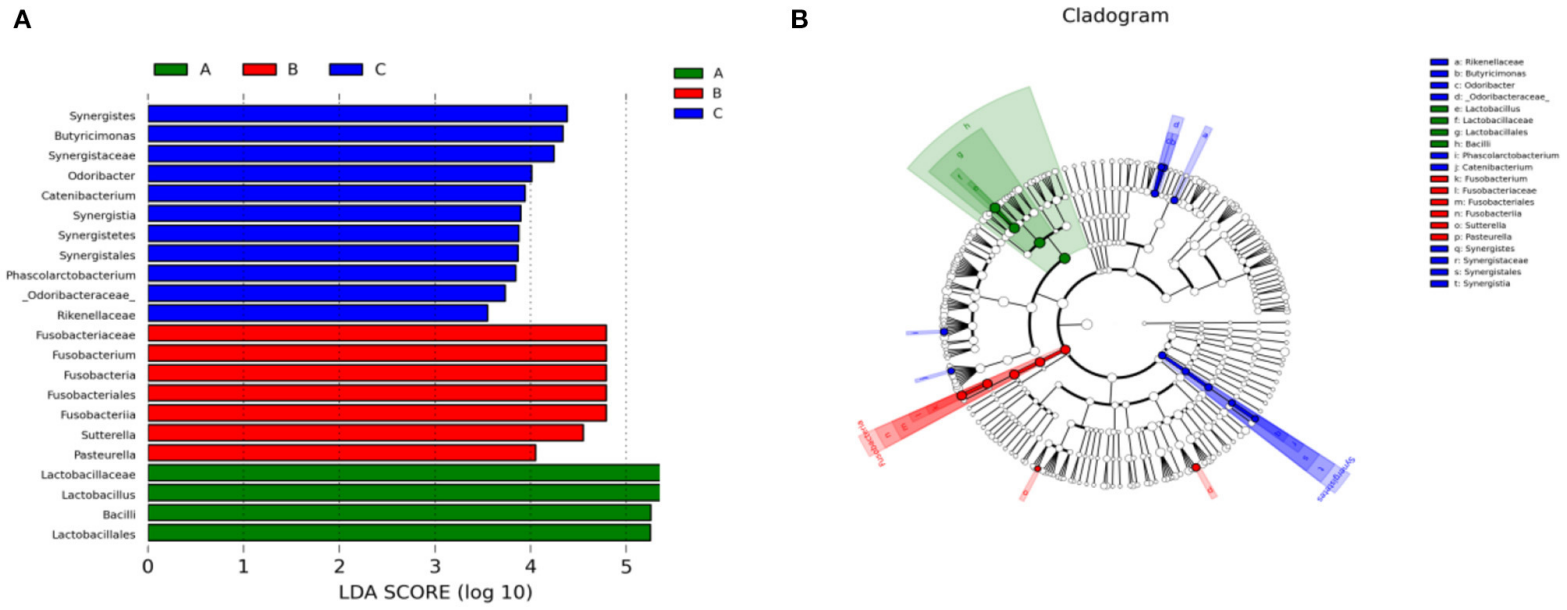
Linear discriminant analysis effect size (LEfSe) analysis of fecal microbiota composition in different groups of Tibetan piglets. **(A)** Histogram of the Linear Discriminant Analysis (LDA) scores computed for bacterial taxa differentially abundant among different groups. **(B)** A cladogram showing statistically and physiologically consistent distinctions among different groups. A: Healthy piglets; B: Diarrheal piglets; C: Treated piglets.

We also performed Linear discriminant analysis effect size (LEfSe) tests to compare further intestinal microflora differences among the three groups (Figure 6). When comparing different Tibetan piglets, we found 4, 7 and 11 bacterial taxa that were abundant in healthy, diarrheal, and treated piglets. Furthermore, healthy piglets had the most enriched phylotypes from the phylum *Lactobacillus*, whereas diarrheal piglets had the most *Sutterella, Fusobacterium*, and *Pasteurella* phlylotype.

## Discussion

Piglet diarrhea is a common issue throughout the pig breeding process. The reasons for piglet diarrhea are quite a lot, such as weaning, nutritional, environmental and physiological stress on piglets (15). In addition, pathogenic bacteria, stress, management factors and excessive feed intake are also associated with piglet diarrhea (16). Moreover, intestinal epithelial mucosal barrier is the first line of defense that animals use to resist in such adverse conditions as it plays an important role in animals' normal intestinal functioning. Therefore, the changes in intestinal microbiota diversity would affect the intestinal function and cause diseases. This study evaluated the fecal microflora structure in healthy, diarrheal and treated piglets of Tibet autonomous region. The findings showed that diarrhea altered the bacterial microbiota structure of Tibetan pigs and impacted the variety of fecal microflora. There were a variety of bacteria in the feces of the Tibetan piglets. By Venn diagram analysis, 1,529 bacterial species were shared among the diarrheal piglets, which were not found in the healthy and treated piglets. Whereas, 2,209 bacterial species were found in the healthy group. PCA analysis showed a significant difference in bacterial community structure among the three groups, especially between healthy and diarrheal piglet groups based on Euclidean distance.

Generally, species are phylogenetically affiliated to phyla Firmicutes, Proteobacteria, and Bacteroidetes, which are abundant in Large White and Chinese Shanxi Black pigs (17). Our results indicated that Firmicutes, Bacteroidetes and Proteobacteria were the most dominant phyla in three groups of Tibetan piglets, which were consistent with previous observations in pig (18), bovine (19), sheep (20) and yak (21). Actinobacteria were mainly distributed in the stomach of herbivores, and they promote fiber decomposition and help in the digestive function of these animals (22). It is noteworthy that Actinobacteria was dominant phylum in Tibetan piglets, which was identified with predecessor's research in wild pigs (23). This phenomenon may be related to the herbivorous nature of Tibetan piglets. Some studies suggest that the abundance of Fusobacteria (24) and Fusobacteria phyla activate host inflammatory responses in order to protect against pathogens that promote tumor growth (25). Remarkably, the higher abundance of Fusobacteria in the fecal microbiota of diarrheal piglets may induce an immune response and increase the risk of pathogen infection of the host. Our results manifested that the Elusimicrobia level in the C group showed an upward trend as compared to the A and B groups, while it was known as an enigmatic bacterial phylum previously. The first representatives were termite gut-associated (26) isolated from the rumen (27) and the environment (28), that comprised of Planctomycetes, Verrucomicrobia, Chlamydia, Omnitrophica, Desantisbacteria (29), Kiritimatiellaeota (30) and Lentisphaerae (31). Cultivation and genome-based studies revealed that some species belonging to Elusimicrobia that are capable of glucose fermentation (32) with the ability to fix nitrogen (28).

*Fusobacterium* is being unveiled pathogen of gastrointestinal disorders. Previous research indicated that *Fusobacterium* plays a role in the pathogenesis of ulcerative colitis (33) and exert potentially carcinogenic (colorectal cancer) effects on the host (34). *Butyricimonas* bacteraemia has been described in patients with colon cancer (35) and patients with posttraumatic chronic bone and joint infections (36). It was isolated from a stool sample of a morbidly obese French patient living in Marseille, using the culturomics approach, which is critical to deciphering the links among gut microbiota and obesity (37). Recent reports link *Sutterella* with gastrointestinal diseases to induce substantial inflammation; rather, it can degrade IgA (38). *Peptostreptococcus* promotes colorectal carcinogenesis and modulates tumor immunity (39).

On the contrary, indole acrylic acid produced by commensal *Peptostreptococcus* species suppresses inflammation (40). Nevertheless, we observed that *Peptostreptococcus* was significantly higher in the diarrheal Tibetan piglets than in the other two groups. Its exact mechanism in Tibetan pigs needs to be further studied. *Pasteurella* are one of the important pathogens that infect a wide range of animals, including swine atrophic rhinitis (41), porcine respiratory disease complex (42), bovine hemorrhagic septicemia (43, 44), avian cholera (45–47) and rabbit respiratory disease (48, 49). A specific decrease in the abundance of *Lactobacillus* in diarrheal Tibetan piglets increased after antibiotic treatment in Tibetan piglets. *Lactobacillus* has been widely recognized for its role in gut microbiota, metabolism, immunity, and health maintenance (50–52).

Additionally, *Lactobacillus* is widely used in animal production because of its antibacterial activity and various biological characteristics (53). *Megasphaera* is a lactate-utilizing bacterium whose ruminal abundance is significantly elevated during fat milk depression (54), producing several short-chain fatty acids (SCFAs). These SCFAs serve as an energy source for host animals and play an important role in gut health (55). The genus *Clavibacter* harbors economically important plant pathogens, infecting crops such as potato and tomato (56, 57). Thus, our results conveyed important information that the relative abundances of pathogenic bacteria (such as *Fusobacterium, Butyricimonas, Sutterella, Peptostreptococcus, Pasteurella*) increased in the diarrheal Tibetan piglets, which disrupted the normal dynamic balance of the intestinal microbiota and led to a competitive decrease in the abundance of beneficial bacteria (*Lactobacillus, Megasphaera*). This phenomenon may also be the main cause of diarrhea in weaned Tibetan piglets. In addition, the intestinal microbial structure was changed by antibiotic treatment in weaned Tibetan piglets. Moreover, abundance of *Lactobacillus* was also increased significantly after antibiotic treatment.

Overall, there were significant difference in gut microbial composition and structure among the groups. Hence, the current study suggested that the decreased relative abundance of beneficial bacteria and increased relative abundance of pathogenic bacteria might cause diarrhea in Tibetan piglets. Therefore, this study provides a better insight into microbial population structure in order to prevent diarrhea in weaned Tibetan piglets.

## Data Availability Statement

The datasets presented in this study can be found in online repositories. The names of the repository/repositories and accession number(s) can be found at: https://www.ncbi.nlm.nih.gov/, PRJNA739650.

## Ethics Statement

Ethical review and approval were not required for the animal study because the present used only fecal samples of Tibetan piglets. Fresh feces were collected by the Animal Care Staff (keepers) during their routine cleaning of the enclosure or directly from the soil without influencing the animals.

## Author Contributions

QK: conceptualization and writing original draft. WZ and MA: methodology. ZS, ZT, and YX: formal analysis and investigation. MK: review and editing. JL and SL: supervision, technical assistance, and funding. All authors participated in the writing of the manuscript, read, and approved the final manuscript.

## Funding

This study was supported by National Natural Science Foundation of China (31760673) and Tibet Autonomous Region Department and College Joint Foundation Project (XZ202101ZR0020G).

## Conflict of Interest

The authors declare that the research was conducted in the absence of any commercial or financial relationships that could be construed as a potential conflict of interest.

## Publisher's Note

All claims expressed in this article are solely those of the authors and do not necessarily represent those of their affiliated organizations, or those of the publisher, the editors and the reviewers. Any product that may be evaluated in this article, or claim that may be made by its manufacturer, is not guaranteed or endorsed by the publisher.
