# Supplementary material for: Characterization of Bacterial Microbiota Composition in Healthy and Diarrheal Early-Weaned Tibetan Piglets
Source: Front Vet Sci. 2022 Feb 23;9:799862. doi: 10.3389/fvets.2022.799862 (PMC8905297; doi:10.3389/fvets.2022.799862)
Supplement: Supplementary file 1 [file Image_1.pdf]

Supplementary Material

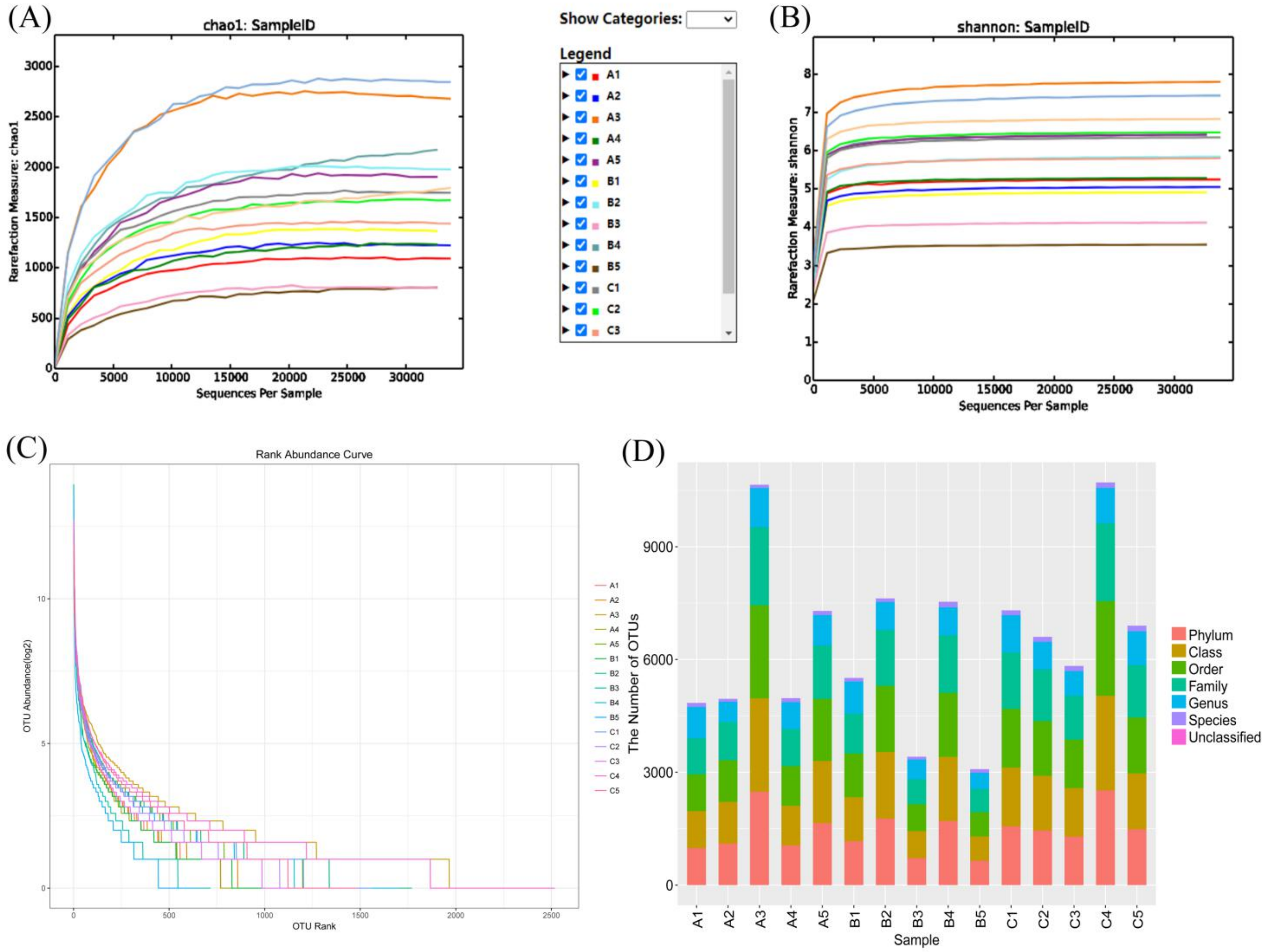

Supplementary figure S1 The Microbial community diversity index curve in different piglet samples (**(A)**: Chao1; **(B)**: Shannon). **(C)** The rank abundance curve of different piglet samples. **(D)** Number of OTUs. **A1-A5**: Healthy piglets; **B1-B5**: Diarrheal piglets; **C1-C5**: Treatment piglets.
